# 

**Published:** 1949-04

**Authors:** 


Bristol

INDEXED

PUBLISHED UNDER THE AUSPICES OF THE BRISTOL MEDICO-CHIRURGICAL SOCIETY

### THE BRISTOL MEDICO-CHIRURGICAL JOURNAL

*A Journal of the Medical Sciences for the*

### WEST OF ENGLAND AND SOUTH WALES

Editor:

E. WATSON-WILLIAMS, *M.C.*, M.D., Ch.M., F.R.C.S.

*with whom are associated*

G. E. F. SUTTON, *M.C.*, M.D., F.R.C.P.

J. M. NAISH, M.D., M.R.C.P.

N. S. CRAIG, *M.C.*, M.D., *Assistant Editor.*

*Local Correspondents:*
BATH—A. D. BATEMAN, *O.B.E.*, F.R.C.S.
BOURNEMOUTH—A. MACKEZIE ROSS, M.D.
CARDIFF—J. D. SPILLANE, M.D., M.R.C.P.
EXETER—Dr. L. N. JACKSON, *M.C.* GLOUCESTER—Dr. R. F. JARRETT, M.B., M.R.C.P.
NEWPORT—J. L. D. WILLIAMS, M.B., F.R.C.S. PLYMOUTH—Dr. C. R. CROFT.
TAUNTON—Dr. M. McALLEY, D.M.R.
TORQUAY—Dr. A. ROBINSON THOMAS, D.M.R.E.
TRURO—Dr. F. D. M. HOCKING, F.R.I.C. YEOVIL—J. A. VERE NICOLL, F.R.C.S.

BRISTOL:
J. W. ARROWSMITH LTD., QUAY STREET AND SMALL STREET

Vol. LXVI. No. 238. *Price:* FOUR SHILLINGS net. APRIL · 1949

